# Variable number of tandem repeat polymorphisms of the interleukin-1 receptor antagonist gene *IL-1RN*: a novel association with the athlete status

**DOI:** 10.1186/1471-2350-11-29

**Published:** 2010-02-22

**Authors:** Sabina Cauci, Manuela Di Santolo, Kelli K Ryckman, Scott M Williams, Giuseppe Banfi

**Affiliations:** 1 Department of Biomedical Sciences and Technologies, School of Medicine, Università di Udine, Udine, Italy; 2 Center for Human Genetics Research, Vanderbilt University, Nashville, TN, USA; 3 Current Address: Department of Pediatrics, University of Iowa, Iowa City, IA, USA; 4 IRCCS, Orthopedic Hospital Galeazzi, and School of Medicine, Università degli Studi di Milano, Milan, Italy

## Abstract

**Background:**

The interleukin-1 (IL-1) family of cytokines is involved in the inflammatory and repair reactions of skeletal muscle during and after exercise. Specifically, plasma levels of the IL-1 receptor antagonist (IL-1ra) increase dramatically after intense exercise, and accumulating evidence points to an effect of genetic polymorphisms on athletic phenotypes. Therefore, the IL-1 family cytokine genes are plausible candidate genes for athleticism. We explored whether IL-1 polymorphisms are associated with athlete status in European subjects.

**Methods:**

Genomic DNA was obtained from 205 (53 professional and 152 competitive non-professional) Italian athletes and 458 non-athlete controls. Two diallelic polymorphisms in the IL-1β gene (*IL-1B*) at -511 and +3954 positions, and a variable number tandem repeats (VNTR) in intron 2 of the IL-1ra gene (*IL-1RN*) were assessed.

**Results:**

We found a 2-fold higher frequency of the *IL-1RN *1/2 genotype in athletes compared to non-athlete controls (OR = 1.93, 95% CI = 1.37-2.74, 41.0% vs. 26.4%), and a lower frequency of the 1/1 genotype (OR = 0.55, 95% CI = 0.40-0.77, 43.9% vs. 58.5%). Frequency of the *IL-1RN *2/2 genotype did not differ between groups. No significant differences between athletes and controls were found for either -511 or +3954 *IL-1B *polymorphisms. However, the haplotype (-511)C-(+3954)T-(VNTR)2 was 3-fold more frequent in athletes than in non-athletes (OR = 3.02, 95% CI = 1.16-7.87). Interestingly, the *IL-1RN *1/2 genotype was more frequent in professional than in non-professional athletes (OR = 1.92, 95% CI = 1.02-3.61, 52.8% vs. 36.8%).

**Conclusions:**

Our study found that variants at the IL-1ra gene associate with athletic status. This confirms the crucial role that cytokine IL-1ra plays in human physical exercise. The VNTR *IL-1RN *polymorphism may have implications for muscle health, performance, and/or recovery capacities. Further studies are needed to assess these specific issues. As VNTR *IL-1RN *polymorphism is implicated in several disease conditions, athlete status may constitute a confounding variable that will need to be accounted for when examining associations of this polymorphism with disease risk.

## Background

Factors that affect athletic performance are only partially defined. However, there are accumulating data indicating that both pro- and anti-inflammatory responses can play a role. Prolonged strenuous exercise results in an activation of the acute phase response with some similarities to sepsis [1]. The induction of a systemic cytokine response, including elevated plasma levels of interleukin (IL)-1 receptor antagonist (IL-1ra), IL-6, IL-8, IL-10, and granulocyte colony-stimulating factor (G-CSF), following intense physical activity, is well documented [1-5]. IL-1ra is a member of the IL-1 family, which includes the classical IL-1 (α and β) cytokines, IL-18 and the newly described IL-1F 5-11 [6]. IL-1ra acts as an antagonist of the IL-1 receptor type I (IL-1RI) and prevents IL-1 (α and β)-dependent signaling. In the absence of IL-1ra, the activity of IL-1 is unopposed, promoting rampant inflammation. Recent research has shown that deficiency of IL-1ra in humans can cause IL-1-mediated systemic and local inflammation that affect infants soon after birth [7]. IL-1α is an IL-1RI agonist with proinflammatory action, that acts primarily as an intracellular transcriptional regulator; it is not found in high concentration in the circulation. In contrast, IL-1β can be secreted from cells in large quantities and thus it is commonly found in serum and secretions. In general, IL-1β acts synergistically with tumor necrosis factor (TNF)-α, activating proinflammatory responses in a wide range of cells and promoting the acute phase response [6]. For these reasons IL-1β levels have been studied in relation to physical activity. However, the circulating concentration of IL-1β is either unaffected by exercise, or it exhibits relatively small, delayed changes [1,2]. In contrast to minimal changes in serum circulating IL-1β after exercise, there is evidence of increased local IL-1β levels after exercise within skeletal muscle; this is most likely associated with response to micro-injury of skeletal muscle caused by intense physical activity [8]. Interestingly, Mahoney and colleagues [9] demonstrated that elevated gene expression of IL-1RI is induced in skeletal muscle after eccentric exercise. Inflammatory and chemoattractant mediators likely promote phagocytosis of trauma-induced cellular debris by macrophages and these cells can continue to secrete IL-1β up to 5 days post-injury [8]. It is of note that accumulation and activation of muscle resident macrophages is a rich source of growth factors postulated to stimulate myogenesis [4]. Thus, inflammation may serve as a mechanism promoting hypertrophy. However, pre- and post-exercise levels of inflammatory factors display considerable variation among people, and this is likely influenced, at least partially, by genetic variation [10,11].

IL-1β plays an important role in the regulation of acute inflammatory response [12]. After an insult to the body, IL-1β is one of the first pro-inflammatory cytokines to be activated. Intense physical activity can cause micro-injury to skeletal muscle, and cytokines such as IL-1β and TNF-α are thought to initiate and regulate the repair process. IL-1β is able to induce the secretion of several inflammatory factors, such as IL-6, IL-8, TNF-α, and GM-CSF, by many different cell types including myoblasts, smooth and skeletal muscle cells, fibroblasts, macrophages, peripheral blood mononuclear cells (PBMC) and endothelial cells [12-14]. IL-1β binds IL-1RI on the surface of a variety of cells and initiates a cascade of events leading to recruitment and activation of macrophages and neutrophils. However, inflammation must be strictly regulated to avoid secondary tissue damage and myopathy. The activity of IL-1β is modulated mainly by a receptor antagonist (IL-1ra) that inhibits IL-1 action by competing for its receptor [6,15]. In healthy individuals, IL-1ra is easily detectable in plasma (concentrations are hundreds of pg/mL), but IL-1β levels are usually undetectable (very few or less than pg/mL) [15,16]. In general, after an insult to the body both IL-1β and IL-1ra increase, and thus, plasma levels of these cytokines are positively correlated [17]. However, the final inflammatory activity of IL-1β is considered to be dependent on the actual ratio of IL-1β over IL-1ra [7,18].

There is evidence that susceptibility to inflammation is influenced by genetic variation in cytokine genes [14,19]. Several studies showed that polymorphisms in the IL-1β (*IL-1B*) and IL-1ra (*IL-1RN*) genes correlate with altered protein expression *in vitro *[14,20] and *in vivo *[10,16,21,22]. These two genes map close to each other on human chromosome 2 [12]. Polymorphisms in the IL-1 gene cluster associate with many clinical conditions, including inflammatory and infectious diseases (periodontal and arterial diseases, altered metabolic conditions, type 2 diabetes, type 2 diabetes complications, gastric cancer, rheumatoid arthritis, ankylosing spondylitis, systemic inflammation, myopathies, Alzheimer's disease, malaria, and bacterial vaginosis) [6,12,17,19,21,23-31]. Mostly, two single nucleotide polymorphisms (SNPs) in *IL-1B *have been studied for disease predisposition, one at position -511 in the promoter region [32] and another at position +3954 in exon 5 (*TaqI *restriction site polymorphism) [18]. In addition, a pentaallelic polymorphic site in intron 2 of the *IL-1RN *gene consisting in a variable number tandem repeats (VNTR, 86 bp repeats) has been extensively investigated in relation to a variety of pathological conditions, including inflammatory myopathies [6,18,19,29,33,34].

Recently, a study performed on 24 sedentary subjects (of unspecified ethnicity) selected on the basis of their haplotype pattern in the IL-1 gene cluster (based on specific combinations of +4845 *IL-1A*, +3954 *IL-1B*, -511 *IL-1B*, and -3737 *IL-1B *polymorphisms) showed that subjects C/C for *IL-1B *+3954 who carry allele 2 at *IL-1RN *+2018 were associated with the inflammation of skeletal muscle, following acute resistance exercise [10].

The aim of this study was to investigate whether polymorphisms in the IL-1β and IL-1ra genes affect athletic status, competitive athlete vs. non-athlete. We evaluated whether the genotype frequencies of -511 and +3954 SNPs of *IL-1B*, and intron 2 VNTR of *IL-1RN *vary between athletes and non-athletes. Athletes of National or Regional competitive standard were recruited. This analysis was performed in a relatively homogeneous ethnic group of Caucasian Italian subjects because associations between polymorphisms in immune system-related genes may be confounded by allele frequency differences between ethnic groups [35,36]. We examined whether the polymorphisms of these genes are associated with being a professional (high-grade) or non-professional (medium-grade) athlete.

## methods

### Subjects and biological samples

The study population consisted of subjects attending the Galeazzi Hospital for routine blood testing. Exclusion criteria were age younger than 18 years and presence of a pathologic condition or chronic major diseases such as diabetes, autoimmune diseases, cardiovascular diseases and malignancies. Athletes were enrolled from teams of Italian professional or non-professional athletes during routine physical examinations. Controls were recruited from the general Italian population of unrelated subjects after the following questions: 1) are you or have you ever been a trained athlete?; 2) did you ever participate in an official athletic competition? All control subjects were without any competitive sport experience, they declared they had never been an athlete or participated in any athletic competition or athletic training. All subjects were Caucasian. The study was approved by the Institutional Review Board of Milan Azienda Sanitaria Locale (ASL). The methods used in this study were in accordance with the Helsinki Declaration of 1975 as revised in 1996. All eligible participants were enrolled consecutively after written informed consent.

Out of 688 subjects enrolled, 25 subjects were excluded from the study because 14 did not fulfill inclusion criteria and 11 had non-interpretable laboratory data. The final analyses were performed on 663 subjects, including 205 (30.9%) athletes and 458 (69.1%) non-athlete controls. The study population of 205 athletes included 53 high-grade professionals (50 current and 3 who had been professional athletes) who compete(d) at National level and 152 medium-grade non-professional athletes who participate in Regional athletic competitions. Specifically, high-grade athletes group included National Italian team grade (some of these athletes participated in Olympic Games, and some were medallists in International Games) or Third Division soccer players. The "caliber" of an Italian Third Division soccer team is similar to National teams of several European countries. The non-professional athlete group included athletes who are highly involved in sports activities (training and competitions >10 h/week).

Among the athletes there were 139 females and 66 males. Age range of study athletes was 18-53 years. A total of 458 healthy Italian non-athlete subjects of both sexes in the age range of 18-53 years (mean 33.6 ± 9.23 years) constituted the control group.

Blood samples were obtained from seated and fasting subjects in the morning from the antecubital vein with evacuated ethylenediamine tetra acetic acid (EDTA) tubes (Vacutainer Tubes, Becton-Dickinson, Franklin Lakes, NJ, USA). Samples were centrifuged at 2000 *g *for 10 min at 4°C and the cellular pellet was processed for DNA extraction. Personnel isolating and processing the DNA were blinded to the subjects' demographic characteristics.

### *IL-1B *and *IL-1RN *polymorphisms gene identification

Genomic DNA was extracted from the blood pellet fraction according to the standard proteinase-K digestion and ethanol extraction method. The extracted DNA was then stored at -20°C until further analysis.

To examine the -511 SNP (rs16944) [37], the promoter region of *IL-1B *was amplified by PCR, using the primers 5'-TGGCATTGATCTGGTTCATC-3' and 5'-GTTTAGGAATCTTCCCACTT-3' as described [32]. The protocol included 35 cycles at 94°C for 1 min, 55°C for 30 s, and 72°C for 30 s, and a final extension at 72°C of 5 min. At the end of the procedure the amplicons were digested with *AvaI *at 37°C for 3 hours. Fragments were analyzed after electrophoresis on 10% acrylamide gels and visualized with ethidium bromide. This gave products of 190 bp and 114 bp (C allele) or 304 bp (T allele) [31]. Out of 663 study subjects, 4 data were not available for the -511 genotype.

To determine the +3954 (rs1143634) [37] SNP genotype, the polymorphic region containing the *TaqI *restriction site (designed as +3953 in older literature [30]) was amplified using the following primers: 5'-GTTGTCATCAGACTTTGACC-3' and 5'-TTCAGTTCATATGGACCAGA-3' [18]. The 249 bp products were digested with *TaqI *at 65°C for 1 hour, resulting in fragments that either remained intact (T allele) or were digested into 2 fragments of 135 and 114 bp (C allele). The restriction fragments were analyzed by electrophoresis on 10% acrylamide gels and visualized with ethidium bromide [31].

The *IL-1RN *intron 2 VNTR polymorphism (rs380092) [38] was analyzed using 5'-CTCAGCAACACTCCTAT-3' and 5'-TCCTGGTCTGCAGGTAA-3' as primers [18,33]. The PCR products of 410 bp (allele 1 = 4 repeats of the 86 bp region), 240 bp (allele 2 = 2 repeats), 500 bp (allele 3 = 5 repeats), 325 bp (allele 4 = 3 repeats), 595 bp (allele 5 = 6 repeats) [18] were analyzed by electrophoresis on 8% acrylamide gel stained with ethidium bromide [31]. A blinded quality control was performed in a random subset of 20 samples, which were sequenced by use of ABI Prism 310 genetic analyzer (Applied Biosystems). The sample consisted of 11 subjects 1/1, 6 subjects 1/2, and 3 subjects 2/2 genotype; a 100% correspondence with gel data was obtained.

### Statistical analysis

The genotype frequencies for each polymorphism were compared by 2-sided Pearson chi-square or Fisher's exact test as appropriate, using STATA statistical software version 10, College Station, TX, USA and Powermarker [39]. The odds ratio (OR) and the 95% confidence interval (CI) were calculated to evaluate the genotype effects of each genotype against all others. Tests for deviations from Hardy-Weinberg equilibrium (HWE) were performed in Powermarker, using a chi-square distribution in cases and controls separately for each SNP. Haplotype analysis was performed with the program Unphased [40]. Haplotypes estimated to be below 1% in frequency were not reported. Due to the low allele frequencies of the 3, 4 and 5 allele of *IL-1RN*, the intron 2 VNTR genotypes were grouped into the short allele (S, allele 2) and the long alleles (L, alleles 1, 3, 4, and 5) according to Machado et al. [41]. Linkage disequilibrium (LD) between the two SNPs in *IL-1B *and the VNTR short and long alleles in *IL-1RN *was determined using Haploview [42]. Differences in the haplotype distributions between cases and controls were determined by likelihood ratio tests. The reference distribution for which all the other haplotypes were compared against was the most common haplotype. The OR was considered significant if the CI did not cross unity. The *P*-value for each haplotype was determined by comparing the haplotype against all others. Therefore, a *P*-value may be slightly significant even if the OR is not.

## Results

The study population consisted of 205 athletes; of these 53 were professional (high-grade, National level) and 152 were non-professional (medium-grade, Regional level, recreational athletes not paid for performing competitions) athletes regularly participating in athletic competitions. Controls were 458 healthy non-athletes. Demographic characteristics and sport disciplines practiced by the 205 athletes are shown in Table 1.

**Table 1:** Demographic characteristics and sport activities of 205 study athletes.

Characteristics	n = 205
Age (years), mean ± SD	25.5 ± 6.68
Body mass index (kg/m^2^), mean ± SD	22.3 ± 3.20
High-grade professional athletes, n (%)	53 (25.9)
Females, n (%)	139 (67.8)^a^
Males, n (%)	66 (32.2)^b^
Volleyball, n (%)	82 (40.0)
Soccer, n (%)	34 (16.6)^c^
Rugby, n (%)	26 (12.7)^d^
Triathlon, n (%)	21 (10.2)^e^
Basketball, n (%)	8 (3.9)
Martial arts, n (%)	6 (2.9)
Track-and field sports, n (%)	6 (2.9)
Running, n (%)	3 (1.5)
Handball, n (%)	3 (1.5)
Swimming, n (%)	3 (1.5)
Others, n (%)	13 (6.3)

^a^4 were professional athletes

^b^48 were professional athletes

^c^22 were professional athletes

^d^23 were professional athletes

^e^8 were professional athletes

A total of 663 study subjects were genotyped for the three gene loci (*IL-1B *in position -511 and +3954; and *IL-1RN *intron 2 VNTR).

Neither case nor control samples deviated from Hardy Weinberg equilibrium (HWE) at site -511 or site +3954. The controls deviated from HWE at the VNTR in *IL-1RN *(*P *= 0.001); while athletes marginally deviated from HWE (*P *= 0.097). Athletes and controls did not differ in the distribution of -511 *IL-1B *genotypes; 38.8% athletes vs. 44.8% controls were homozygous CC, 48.8% athletes vs. 45.9% controls were heterozygous CT, and 12.4% athletes vs. 9.4% controls were homozygous TT (Table 2). The two groups also did not differ significantly in allele frequency (*P *= 0.11).

**Table 2:** Genotype frequencies of *IL-1B *promoter at position -511 in 659 white subjects, comparison of 201 athletes and 458 non-athlete controls.

	All subjects (n = 659)	Athletes (n = 201)	Controls (n = 458)	Odds ratio (95% CI)	*P*-value Athletes versus controls
*IL-1B *promoter genotype (-511)					0.264
CC	283 (43.0%)	78 (38.8%)	205 (44.8%)	0.78 (0.56-1.10)	0.155
CT	308 (46.7%)	98 (48.8%)	210 (45.9%)	1.12 (0.81-1.57)	0.491
TT	68 (10.3%)	25 (12.4%)	43 (9.4%)	1.37 (0.81-2.31)	0.236
					
*IL-1B *promoter allele					0.111
Allele C	874 (66.3%)	254 (63.2%)	620 (67.7%)		
Allele T	444 (33.7%)	148 (36.8%)	296 (32.3%)		

Genotype and allele distribution of *IL-1B *+3954 gene polymorphism are shown in Table 3. Athletes and controls did not differ; 60.0% athletes vs. 62.2% controls were homozygous CC, 36.1% athletes vs. 32.3% controls were heterozygous CT, and 3.9% athletes vs. 5.5% controls were homozygous TT. The two groups also did not differ significantly in allele frequency (*P *= 0.89).

**Table 3:** Genotype frequencies of *IL-1B *exon 5 at position +3954 in 663 subjects, comparison of 205 athletes and 458 non-athlete controls.

	All subjects (n = 663)	Athletes (n = 205)	Controls (n = 458)	OR (95% CI)	*P*-value Athletes versus controls
*IL-1B *exon 5 genotype (+3954)					0.495
CC	408 (61.5%)	123 (60.0%)	285 (62.2%)	0.91 (0.65-1.28)	0.586
CT	222 (33.5%)	74 (36.1%)	148 (32.3%)	1.18 (0.84-1.67)	0.340
TT	33 (5.0%)	8 (3.9%)	25 (5.5%)	0.70 (0.31-1.59)	0.395
					
*IL-1B *exon 5 allele					0.891
Allele C	1038 (78.3%)	320 (78.0%)	718 (78.4%)		
Allele T	288 (21.7%)	90 (22.0%)	198 (21.6%)		

For the *IL-1RN *VNTR polymorphism three alleles (allele 1, 2 and 3) were common in our study; allele 4 was found in 3 subjects (one genotype 1/4 and two genotypes 2/4, all in the control group) and allele 5 was detected in only 1 subject (one genotype 1/5, in the athlete group). The most common allele, the IL-1RN*1 allele, was less frequent in athletes than in controls (65.4% vs. 74.0%, *P *= 0.001), whereas the second commonest allele, IL-1RN*2, had an allele frequency higher in athletes than controls (32.2% vs. 22.9%, *P *< 0.001), finally, IL-1RN*3 had similar frequency in athletes and controls (*P *= 0.57) (overall alleles, *P *= 0.001) (Table 4). Specifically, athletes were less likely to have the 1/1 genotype (OR = 0.55, 95% CI 0.40-0.77), and 2-fold more likely to be 1/2 (OR = 1.93, 95% CI 1.37-2.74). Athletes and controls did not differ in frequency of the 2/2 genotype. However, we observed increased frequencies of cumulative 1/2, 2/2, 2/3 and 2/4 genotypes in athletes (53.7%) compared with controls (36.7%), OR = 2.00 (95% CI = 1.43-2.79), suggesting a dominant effect of the 2 allele. These observations were significant in both male and female athletes when analyzed separately (data not shown, see additional File 1). Genotypes that included the 3 allele did not differ between athletes and controls.

**Table 4:** Genotype frequencies of *IL-1RN *VNTR, comparison of 205 athletes and 458 non-athlete controls.

	All subjects (n = 663)	Athletes (n = 205)	Controls (n = 458)	OR (95% CI)	*P*-value Athletes versus controls
*IL-1RN *VNTR genotype					**< 0.001**
1/1	358 (54.0%)	90 (43.9%)	268 (58.5%)	**0.55 (0.40-0.77)**	**< 0.001**
1/2	205 (30.9%)	84 (41.0%)	121 (26.4%)	**1.93 (1.37-2.74)**	**< 0.001**
1/3	23 (3.5%)	3 (1.5%)	20 (4.4%)	0.33 (0.10-1.11)	0.067
2/2	64 (9.7%)	22 (10.7%)	42 (9.2%)	1.19 (0.69-2.05)	0.529
2/3	7 (1.1%)	4 (2.0%)	3 (0.7%)	3.02 (0.67-13.6)	0.211
1/2 and 2/2 and 2/3 and 2/4	278 (41.9%)	110 (53.7%)	168 (36.7%)	**2.00 (1.43-2.79)**	**< 0.001**
1/3 and 2/3 and 3/3	32 (4.8%)	8 (3.9%)	24 (5.2%)	0.73 (0.32-1.66)	0.458
					
*IL-1RN *VNTR allele					**0.001**
Allele 1	946 (71.3%)	268 (65.4%)	678 (74.0%)	**0.66 (0.52-0.85)**	**0.001**
Allele 2	342 (25.8%)	132 (32.2%)	210 (22.9%)	**1.60 (1.23-2.07)**	**< 0.001**
Allele 3	34 (2.6%)	9 (2.2%)	25 (2.7%)	0.80 (0.37-1.73)	0.570

In a secondary analysis (Table 5), we compared *IL-1RN *genotypes between the 53 professional athletes and the 152 non-professional athletes. Interestingly, we found that the 1/2 VNTR genotype was almost 2-fold more frequent in professional than in non-professional athletes (OR = 1.92, 95% CI = 1.02-3.61). No other significant differences were noted between professional and recreational athletes.

**Table 5:** Genotype frequencies of *IL-1RN *VNTR comparison of 53 professional athletes and 152 non-professional athletes and 458 non-athlete controls.

	Professional Athletes (n = 53)	Non-professional Athletes (n = 152)	OR^a^ (95% CI)	*P*-value^a^	OR^b^ (95% CI)	*P*-value^b^	OR^c^ (95% CI)	*P*-value^c^
*IL-1RN *VNTR genotype				0.084		**< 0.001**		**0.047**
1/1	19 (35.8%)	71 (46.7%)	0.64 (0.33-1.22)	0.200	**0.40 (0.22-0.72)**	**0.002**	**0.62 (0.43-0.90)**	**0.012**
1/2	28 (52.8%)	56 (36.8%)	**1.92 (1.02-3.61)**	**0.042**	**3.12 (1.75-5.56)**	**< 0.001**	**1.62 (1.10-2.40)**	**0.014**
1/3	0	3 (2.0%)	0.98 (0.96-1.00)	0.570	0.96 (0.94-0.98)	0.250	0.44 (0.13-1.51)	0.225
2/2	3 (5.7%)	19 (12.5%)	0.42 (0.12-1.48)	0.205	0.59 (0.18-1.99)	0.607	1.41 (0.80-2.52)	0.236
2/3	2 (3.8%)	2 (1.3%)	2.94 (0.40-21.4)	0.275	5.95 (0.97-36.4)	0.086	2.02 (0.33-12.2)	0.603
1/2 and 2/2 and 2/3 and 2/4	33 (62.3%)	77 (50.7%)	1.61 (0.85-3.05)	0.145	**2.84 (1.58-5.12)**	**< 0.001**	**1.77 (1.22-2.57)**	**0.002**
1/3 and 2/3 and 3/3	2 (5.2%)	6 (3.7%)	0.95 (0.19-4.88)	1.000	0.71 (0.16-3.09)	1.000	0.74 (0.30-1.85)	0.523
								
*IL-1RN *VNTR allele				0.498		**0.019**		**0.023**
Allele 1	67 (63.2%)	201(66.1%)						
Allele 2	36 (34.0%)	96 (31.6%)						
Allele 3	2 (1.9%)	7 (2.3%)						

^a^Comparison between professional and non-professional athletes

^b^Comparison between professional athletes and non-athlete controls

^c^Comparison between non-professional athletes and non-athlete controls

Comparison of professional athletes with non-athletes revealed that genotype 1/2 VNTR was 3-fold more frequent in high-grade athletes (OR = 3.12, 95% CI = 1.75-5.56) (Table 5). The same genotype was only 1.6-fold more frequent in medium grade/recreational athletes than in non-athletes (OR = 1.62, 95% CI = 1.10-2.40) (Table 5). The 1/1 genotype was 2.5-fold more frequent in non-athletes than in professional athletes (OR = 2.52, 95% CI = 1.40-4.56), but 1.6-fold more frequent in non-athletes than in recreational athletes (OR = 1.61, 95% CI = 1.11-2.33). Overall (frequencies already reported in Table 4), the 1/1 genotype was 1.8-fold more frequent in non-athletes than in all athletes (OR = 1.80, 95% CI = 1.29-2.51).

**Figure 1:**
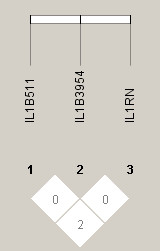
**Linkage disequilibrium plots (r^2^) are shown for athletes**.

**Figure 2:**
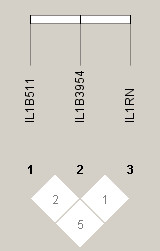
**Linkage disequilibrium plots (r^2^) are shown for non-athletes**.

Haplotype analyses were performed, using all three polymorphisms (Table 6). There was virtually no LD between any of the three sites in athletes (Figure 1) or non-athletes (r^2 ^≤ 0.05) (Figure 2). The three site haplotype was differentially distributed between athletes and controls (*P *= 0.012). Individually only one haplotype associated with athlete status. Specifically, (-511)C-(+3954)T-(VNTR)2 was 3 times more common in athletes than non-athletes (OR = 3.02, 95%CI = 1.15-7.87). We also analyzed the three pair wise haplotypes and found that only those that included the VNTR were significant, indicating that the association was related to the VNTR or a marker in linkage disequilibrium with it.

**Table 6:** Haplotype frequencies in athletes and non-athlete controls.

	Athletes (n = 205)	Controls (n = 458)	OR (95% CI)	*P*-value
Haplotype (3 sites) -511, +3954, *IL-1RN*)^a^	Haplotype Frequency	Haplotype Frequency		**0.012**
C-C-L	0.360	0.406	1.0 (reference)	**0.029**
C-C-S	0.117	0.093	1.420 (0.862-2.339)	0.095
C-T-L	0.106	0.160	0.745 (0.436-1.274)	0.051
C-T-S	0.049	0.018	**3.016 (1.156-7.867)**	**0.011**
T-C-L	0.171	0.178	1.082 (0.711-1.648)	0.838
T-C-S	0.133	0.107	1.400 (0.891-2.200)	0.054
T-T-L	0.042	0.027	1.762 (0.692-4.487)	0.484
T-T-S	0.022	0.011	2.226 (0.492-10.080)	0.043
Haplotype (2 sites)				
				
-511, +3954				0.264
C-C	0.478	0.498	1.0 (reference)	0.278
C-T	0.154	0.179	0.896 (0.608-1.321)	0.520
T-C	0.303	0.286	1.105 (0.815-1.498)	0.258
T-T	0.065	0.038	1.809 (0.889-3.679)	0.103
				
+3954, *IL-1RN*				**0.001**
C-L	0.529	0.584	1.0 (reference)	**0.023**
C-S	0.251	0.200	**1.386 (1.013-1.897)**	**0.012**
T-L	0.149	0.187	0.879 (0.597-1.292	0.183
T-S	0.071	0.029	**2.658 (1.345-5.254)**	**0.003**
				
-511, *IL-1RN*				**0.003**
C-L	0.465	0.566	1.0 (reference)	**0.001**
C-S	0.166	0.111	**1.821 (1.213-2.732)**	**0.014**
T-L	0.214	0.205	1.267 (0.894-1.795)	0.954
T-S	0.155	0.118	**1.589 (1.099-2.298)**	**0.025**

^a^Due to the low allele frequencies of the 3, 4 and 5 alleles of the intron 2 VNTR *IL-1RN *genotypes were grouped into the short allele (S, allele 2) and the long alleles (L, alleles 1, 3, 4, and 5).

## Discussion

### Polymorphisms and exercise

Our finding that athletes have significantly different genotypes than non-athletes reinforces the evidence that athletic performance is associated with genetic constitution in addition to environmental/behavioral factors. However, we want to emphasize that carriage of a specific genetic polymorphism is only a predisposing factor and does not by itself necessarily result in elite performance. Clearly, more than one biologic process is necessary for the development of high-grade athletic performance.

In general, genetic associations of athletic phenotype are only partially understood. Increased knowledge in this research may help to identify physiological and biochemical pathways related to exercise [43]. Genetic studies so far focused on candidate genes (over 200) mainly involved in cardiovascular performance, including angiotensin-converting enzyme (ACE) and proteins involved in skeletal-muscle activity such as α-actinins [44-46]. Immunity-related genes have been minimally explored [45]. A study that examined polymorphisms of the IL-15 receptor-α (*IL-15RA*) gene identified a SNP strongly associated with muscle hypertrophy in young subjects (76 men and 77 women) of mixed ethnicity who performed resistance exercise training [47]. Two studies performed in non-athletes reported that the -174G/C variant in the IL-6 gene affected the response to exercise by modulating changes in serum concentrations of IL-6 [48] and high-density lipoprotein cholesterol (HDL-C) [49]. However, Walston and colleagues [50] identified no significant relationship between IL-6 SNPs or IL-6 haplotypes and serum IL-6 concentration, grip, knee, or hip strength, or frailty in 463 older women.

### IL-1β and IL-1ra roles in human disease and exercise

The inflammatory response after intensive exercise is a well documented phenomenon; the coordinated action of cytokines is considered crucial for regulation of inflammatory reactions responsible for immune defenses and muscle repair. Conceivably, a delay in the removal of dead cells increases the likelihood of a slow recovery.

IL-1β is a crucial proinflammatory cytokine involved in initiating and amplifying the inflammatory response; it stimulates the production of prostaglandins and nitric oxide, both of which are highly inflammatory, and it induces the synthesis of several chemokines [6,12]. IL-1β is a highly active pleiotropic cytokine with several known functions. It has many systemic effects in the protection of the body, including response to stress and the metabolism of insulin, lipids and bone [12]. Injecting a few nanograms per kilogram of IL-1β results in an increase of acute phase proteins, plasma IL-6, neutrophilia and thrombocytosis [12]. Recent evidence suggests that IL-1β plays a key pathogenic role in many human conditions, including cardiovascular diseases [23,51], and in the central nervous system effects of exercise [52,53]. The bioactivity of IL-1β is finely modulated as it is a potentially harmful factor able to promote tissue damage and fever. The main cytokine that counteracts the effects of IL-1β is IL-1ra, a naturally occurring inhibitor of IL-1β that binds to IL-1 receptor type I, but does not elicit post-receptor signaling [7]. In addition, IL-1ra seems to modulate the IL-1 decoy receptor, IL-1 receptor type II [54]. IL-1ra protein is produced by many cells that can synthesize IL-1β, mainly by hepatocytes, adipocytes, neutrophils and macrophages. The IL-1ra plasma levels are co-coordinately regulated by both the *IL-1RN *and *IL-1B *genes [16,22]. The biological functions of IL-1ra are not completely understood. Interestingly, at present a recombinant IL-1ra (anakinra) is approved for use in humans as an anti-inflammatory therapy in several conditions, such as rheumatic diseases, ankilosing spondylitis, chronic Achilles tendinopathy, type 2 diabetes mellitus and systemic inflammatory diseases [6,7,51,55].

Several studies found that serum IL-1β concentration does not change after physical exercise, whereas IL-1ra concentration significantly increases [1,5]. In this respect, the profile of systemic cytokines' changes in response to exercise differs from that observed in sepsis, where there is a relevant increase in both serum IL-1β and IL-1ra [1]. The rise of circulating anti-inflammatory cytokines induced by exercise has been suggested as one of the pathways involved in protection against several diseases, primarily against cardiovascular disease and type 2 diabetes mellitus exerted by regular exercise [1].

### VNTR polymorphism in the intron 2 of the *IL-1RN *gene

We demonstrated that the genotype -511 in the promoter region and +3954 in exon 5 of *IL-1B *gene have no influence on the athletic phenotype. These findings seem to fit with the *in vivo *observation that neither circulating IL-1β, nor IL-1β mRNA levels are significantly changed by physical activity [1,9,56].

In contrast, we showed that a specific genotype (1/2) of the IL-1ra gene, a VNTR in intron 2 of *IL-1RN*, is almost 2-fold more frequent in athletes than in non-athletes. Additionally, we observed a dose-effect relationship with the 1/2 *IL-1RN *genotype being 2-fold more frequent in professional than in recreational athletes and 3-fold more frequent in professional athletes than in non-athletes. Thus, our study suggests the 1/2 *IL-1RN *frequency is increasing in parallel with the level of athletic grade. We did not observe gender effects.

The precise molecular effects of the *IL-1RN *polymophisms are not totally clear [22,27]. Possibly, the 1/2 *IL-1RN *genotype could favor muscle repair and hypertrophy in athletes; however, further studies are needed to clarify this issue [4,47]. This can occur through an influence on circulating or local levels of IL-1ra in humans [22,27]. Consistent with this is that the IL-1RN*2 allele is associated with a higher production of inflammatory reactions, and therefore, an overall increase of IL-1 activity, meaning less effective IL-1ra inhibitory activity [19,27]. However, contrasting findings were obtained for the association of IL-1RN*2 with IL-1ra levels; some authors found that the IL-1RN*2 allele is associated with reduced IL-1ra [14,57,58], but other authors observed no association [29] or even increased IL-1ra [16,18,21]. Some of these discrepancies can be due to different experimental designs and/or by the presence of heterogeneity with other polymorphisms in the *IL-1RN *gene [27].

In our study athletes were not more likely to be homozygous for the allele 2, a genotype that has been associated with several pathological conditions of inflammatory or autoimmune nature [18,19,26,28,34,59,60]. It is of note that the *IL-1RN *2/2 genotype was more likely associated with disease than the *IL-1RN *1/2, especially for gastric cancer development [19,34,41]. Based on the *in vitro *observation that VNTR allele 2 has been associated with a decreased production of IL-1ra [14,58]; and increased production of IL-1β [20] in a dose-dependent fashion, these findings, taken together, suggest that a moderate increase of IL-1-dependent inflammation, as conferred by one *IL-1RN *allele 2 can favor athletic performance, but not an excessive increase as conferred by the *IL-1RN *2/2 genotype.

It is known that there are different IL-1 gene cluster genetic profiles between ethnic groups, and particularly between black and white populations [35]. Therefore, we considered only white Italian subjects, a homogeneous Caucasian ethnic group. A limitation of this approach is that our results may not be applicable to non-Caucasian subjects.

## Conclusions

Our study supports the notion that the IL-1 family of genes is significantly associated with physical performance. The finding of a higher frequency of allele 2, and specifically of the 1/2 genotype VNTR *IL-1RN*, in athletes with respect to non-athletes highlights that immune genetic variants are involved in athletic predisposition. Based on previous evidence of inflammatory effects of the VNTR *IL-1RN *allele 2, the association of IL-1 related genes with athlete status appears to associate a genetic predisposition of a moderately enhanced inflammatory response. However, we cannot exclude much more indirect effects modulated by IL-1ra. Immunity in physical exercise is likely regulated by subtle tuning of cytokine levels; for example, a partial reduction in IL-1ra could reinforce immune defenses against exercise-associated micro-injuries.

It was beyond the scope of this study to investigate the effects of IL-1 polymorphisms on exercise-induced systemic and local inflammation, recovery capacity and type of sport activity (aerobic vs. anaerobic; endurance vs. sprint), which should be clarified in future studies. Of major interest will be the comparison of aerobic vs. anaerobic athletes; however, in the present study this kind of evaluation was not possible due to small number of athletes participating in purely anaerobic sports. Future studies relating *IL-1RN *genotypes and cytokine levels should focus preferably on immune genetic profiles and inflammatory markers evaluated before and after exercise. Although our results indicate an association between the IL-1 family of cytokines and athlete status, it is not clear how, specifically, the VNTR *IL-1RN *polymorphism affects the phenotype promoting a relationship between this allele and grade of athletic performance.

In this study we focused on 2 SNPs of the *IL-1B *gene and 1 VNTR in the *IL-1RN *gene because these are the most frequently studied polymorphisms of the IL-1β and IL-1ra cytokines, which have been associated with a variety of human conditions. Interestingly, athlete status may constitute a confounding factor contributing to variability of results concerning the association of the *IL-1RN *polymorphisms with pathologic conditions [61].

Our present results should prompt larger studies on additional polymorphisms in the IL-1 family in relation to physical performance and elite athletic grade, particularly, those located in the IL-1ra gene, as the SNP at position +2018.

In conclusion, we demonstrated in a cross-sectional study that a polymorphism potentially able to modulate the levels of the IL-1ra cytokine, and indirectly IL-1 (α and β) activity, is associated with athlete status. Further research into immune factors that increase athletic performance will help to inform the design of how immune modulators may improve recovery or implement cure interventions after injuries. Our finding suggests that clinical trials performed to assess efficacy of various treatments for athlete's recovery should probably differentiate subjects on a genetic basis in addition to etiology to maximize the chances of observing statistically significant and reproducible results. Our study suggests cytokines are important molecules to further explore in relation to sports activity. Overall, this study may contribute to an increased understanding of the genetic basis of athletic performance. It may also increase our understanding of inflammatory mechanisms associated with intensive exercise.

## Competing interests

The authors declare that they have no competing interests.

## Authors' contributions

Conceived and designed the experiments: SC. Performed the experiments: MDS, SC. Analyzed the data and performed statistical analysis: KKR, SMW. Wrote the paper: SC, SMW, GB. All authors read and approved the final manuscript.

## Pre-publication history

The pre-publication history for this paper can be accessed here:

http://www.biomedcentral.com/1471-2350/11/29/prepub

## Supplementary Material

Additional file 1 **Table 7 Genotype frequencies of *IL-1B *promoter at position -511 in female and male athletes. Table 8 Genotype frequencies of *IL-1B *exon 5 at position +3954 in female and male athletes. Table 9 Genotype frequencies of *IL-1RN *VNTR in female and male athletes**. Frequencies of *IL-1B *and *IL-1RN *genotype frequencies stratified by athlete gender. Click here for file
