# Supplementary material for: Variable number of tandem repeat polymorphisms of the interleukin-1 receptor antagonist gene IL-1RN: a novel association with the athlete status
Source: BMC Med Genet. 2010 Feb 22;11:29. doi: 10.1186/1471-2350-11-29 (PMC2837019; doi:10.1186/1471-2350-11-29)
Supplement: Additional file 1 — Table 7 Genotype frequencies of IL-1B promoter at position -511 in female and male athletes. Table 8 Genotype frequencies of IL-1B exon 5 at position +3954 in female and male athletes. Table 9 Genotype frequencies of IL-1RN VNTR in female and male athletes. Frequencies of IL-1B and IL-1RN genotype frequencies stratified by athlete gender. [file 1471-2350-11-29-S1.DOC]

**Table 7** **Genotype frequencies of *IL-1B* promoter at position -511 in female and male athletes.**

|  | **All subjects**  **(n = 593)** | **Female**  **Athletes**  **(n = 135)** | **Non-athlete Controls**  **(n = 458)** | **Odds ratio**  **(95% CI)** | ***P-*value** |
| --- | --- | --- | --- | --- | --- |
| *IL-1B* promoter genotype (-511) |  |  |  |  | 0.175 |
| CC | 256 (43.2%) | 51 (37.8%) | 205 (44.8%) |  |  |
| CT | 275 (46.4%) | 65 (48.1%) | 210 (45.9%) |  |  |
| TT | 62 (10.5%) | 19 (14.1%) | 43 (9.4%) |  |  |
|  |  |  |  |  |  |
| *IL-1B* promoter allele |  |  |  |  | 0.075 |
| Allele C | 787 (66.4%) | 167 (61.9%) | 620 (67.7 %) |  |  |
| Allele T | 399 (33.6%) | 103 (38.1%) | 296 (32.3%) |  |  |

|  | **All subjects**  **(n = 524)** | **Male**  **Athletes**  **(n = 66)** | **Non-athlete Controls**  **(n = 458)** | **Odds ratio**  **(95% CI)** | ***P-*value** |
| --- | --- | --- | --- | --- | --- |
| *IL-1B* promoter genotype (-511) |  |  |  |  | 0.814 |
| CC | 232 (44.3%) | 27 (40.9%) | 205 (44.8%) |  |  |
| CT | 243 (46.4%) | 33 (50.0%) | 210 (45.9%) |  |  |
| TT | 49 (9.4%) | 6 (9.1%) | 43 (9.4%) |  |  |
|  |  |  |  |  |  |
| *IL-1B* promoter allele |  |  |  |  | 0.684 |
| Allele C | 707 (67.5%) | 87 (65.9%) | 620 (67.7 %) |  |  |
| Allele T | 341 (32.5%) | 45 (34.1%) | 296 (32.3%) |  |  |

**Table 8 Genotype frequencies of *IL-1B* exon 5 at position +3954 in female and male athletes.**

|  | **All subjects (n=597)** | **Female**  **Athletes (n=139)** | **Non-athlete Controls (n=458)** | **OR**  **(95% CI)** | ***P-*value** |
| --- | --- | --- | --- | --- | --- |
| *IL-1B* exon 5 genotype (+3954) |  |  |  |  | 0.668 |
| CC | 368 (61.6%) | 83 (59.7%) | 285 (62.2%) |  |  |
| CT | 198 (33.2%) | 50 (36.0%) | 148 (32.3%) |  |  |
| TT | 31 (5.2%) | 6 (4.3%) | 25 (5.5%) |  |  |
|  |  |  |  |  |  |
| *IL-1B* exon 5 allele |  |  |  |  | 0.808 |
| Allele C | 934 (78.2%) | 216 (77.7%) | 718 (78.4%) |  |  |
| Allele T | 260 (21.8%) | 62 (22.3%) | 198 (21.6%) |  |  |

|  | **All subjects (n=524)** | **Male**  **Athletes (n=66)** | **Non-athlete Controls (n=458)** | **OR**  **(95% CI)** | ***P-*value** |
| --- | --- | --- | --- | --- | --- |
| *IL-1B* exon 5 genotype (+3954) |  |  |  |  | 0.643 |
| CC | 325 (62.0%) | 40 (60.6%) | 285 (62.2%) |  |  |
| CT | 172 (32.8%) | 24 (36.4%) | 148 (32.3%) |  |  |
| TT | 27 (5.2%) | 2 (3.0%) | 25 (5.5%) |  |  |
|  |  |  |  |  |  |
| *IL-1B* exon 5 allele |  |  |  |  | 0.916 |
| Allele C | 822 (78.4%) | 104 (78.8%) | 718 (78.4%) |  |  |
| Allele T | 226 (21.6%) | 28 (21.2%) | 198 (21.6%) |  |  |

**Table 9 Genotype frequencies of *IL-1RN* VNTR in female and male athletes*.***

|  | **All subjects (n=597)** | **Female**  **Athletes (n=139)** | **Non-athlete Controls (n=458)** | **OR**  **(95% CI)** | ***P-*value** |
| --- | --- | --- | --- | --- | --- |
| *IL-1RN* VNTR genotype |  |  |  |  | **0.044** |
| 1/1 | 332 (55.7%) | 64 (46.0%) | 268 (58.5%) | **0.60 (0.41-0.89)** | **0.010** |
| 1/2 | 171 (28.7%) | 50 (36.0%) | 121 (26.4%) | **1.56 (1.04-2.34)** | **0.029** |
| 1/3 | 23 (3.9%) | 3 (2.2%) | 20 (4.4%) | 0.48 (0.14-1.65) | 0.318 |
| 2/2 | 61 (10.2%) | 19 (13.7%) | 42 (9.2%) | 1.57 (0.88-2.80) | 0.125 |
| 2/3 | 5 (0.8%) | 2 (1.4%) | 3 (0.7%) | 2.21 (0.37-13.39) | 0.331 |
| 1/2 and 2/2 and 2/3 and 2/4 | 239 (40.1%) | 71 (51.1%) | 168 (36.7%) | **1.80 (1.23-2.64)** | **0.002** |
| 1/3 and 2/3 and 3/3 | 30 (5.0%) | 6 (4.3%) | 24 (5.2%) | 0.82 (0.33-2.04) | 0.662 |
|  |  |  |  |  |  |
| *IL-1RN* VNTR allele |  |  |  |  | **0.012** |
| Allele 1 | 859 (71.9%) | 181 (65.1%) | 678 (74.0%) | **0.66 (0.49-0.87)** | **0.004** |
| Allele 2 | 300 (25.1%) | 90 (32.4%) | 210 (22.9%) | **1.61 (1.20-2.16)** | **0.002** |
| Allele 3 | 32 (2.7%) | 7 (2.5%) | 25 (2.7%) | 0.92 (0.39-2.15) | 0.849 |
|  | **All subjects (n=524)** | **Male**  **Athletes (n=66)** | **Non-athlete Controls (n=458)** | **OR**  **(95% CI)** | ***P-*value** |
| *IL-1RN* VNTR genotype |  |  |  |  | **< 0.001** |
| 1/1 | 294 (56.1%) | 26 (39.4%) | 268 (58.5%) | **0.46 (0.27-0.78)** | **0.003** |
| 1/2 | 155 (29.6%) | 34 (51.5%) | 121 (26.4%) | **2.96 (1.75-5.00)** | **<0.001** |
| 1/3 | 20 (3.8%) | 0 (0.0%) | 20 (4.4%) | - | 0.158 |
| 2/2 | 45 (8.6%) | 3 (4.5%) | 42 (9.2%) | 0.47 (0.14-1.57) | 0.344 |
| 2/3 | 5 (1.0%) | 2 (3.0%) | 3 (0.7%) | 4.74 (0.78-28.91) | 0.122 |
| 1/2 and 2/2 and 2/3 and 2/4 | 207 (39.5%) | 39 (59.1%) | 168 (36.7%) | **2.49 (1.47-4.22)** | **0.001** |
| 1/3 and 2/3 and 3/3 | 26 (5.0%) | 2 (3.0%) | 24 (5.2%) | 0.57 (0.13-2.45) | 0.760 |
|  |  |  |  |  |  |
| *IL-1RN* VNTR allele |  |  |  |  | **0.036** |
| Allele 1 | 765 (73.0%) | 87 (65.9%) | 678 (74.0%) | **0.68 (0.46-1.00)** | **0.050** |
| Allele 2 | 252 (24.0%) | 42 (31.8%) | 210 (22.9%) | **1.57 (1.05-2.33)** | **0.025** |
| Allele 3 | 27 (2.6%) | 2 (1.5%) | 25 (2.7%) | 0.55 (0.13-2.34) | 0.564 |
